# Long non-coding RNA UICLM promotes colorectal cancer liver metastasis by acting as a ceRNA for microRNA-215 to regulate ZEB2 expression: Erratum

**DOI:** 10.7150/thno.108968

**Published:** 2025-01-09

**Authors:** Dong-liang Chen, Yun-xin Lu, Jia-xing Zhang, Xiao-li Wei, Feng Wang, Zhao-lei Zeng, Zhi-zhong Pan, Yun-fei Yuan, Feng-hua Wang, Helene Pelicano, Paul J. Chiao, Peng Huang, Dan Xie, Yu-hong Li, Huai-qiang Ju, Rui-hua Xu

**Affiliations:** 1 State Key Laboratory of Oncology in South China, Collaborative Innovation Center for Cancer Medicine, Sun Yat-sen University Cancer Center, Guangzhou, PR China;; 2 Department of Oncology, The First Affiliated Hospital, Sun Yat-sen University, Guangzhou, PR China;; 3 Department of Molecular and Cellular Oncology, the University of Texas MDAnderson Cancer Center, Houston, TX77030, USA.

The authors regret that some incorrect representative images were accidentally used in our previously published paper, there was one misplaced image in Figure 3A, Figure 5F and Figure S4D, respectively, the corrected versions are provided below. The correction made in this erratum does not affect the original conclusions. The authors apologize for any inconvenience or misunderstanding that this error may have caused.

## Figures and Tables

**Figure A:**
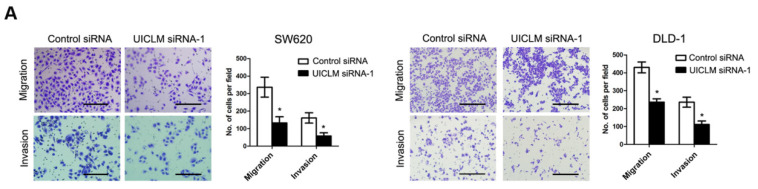
Correct image of Figure 3A

**Figure B:**
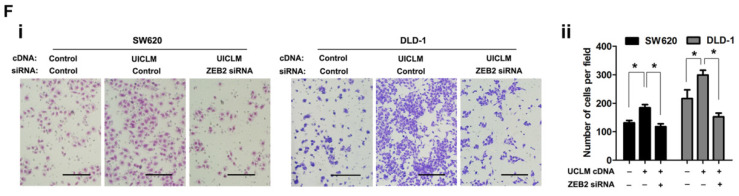
Correct image of Figure 5F

**Figure C:**
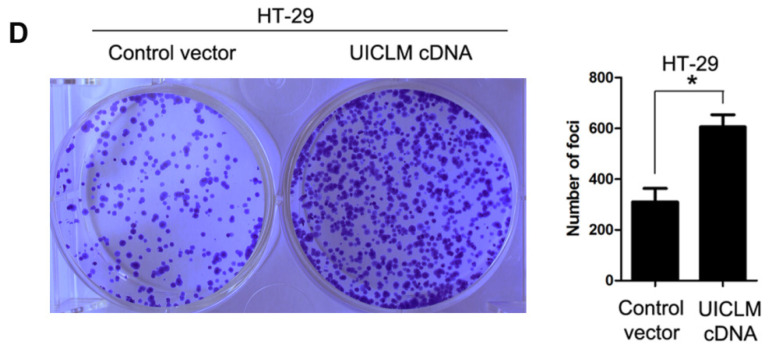
Correct image of Supplementaty Figure 4D

